# Using the IFASIS (Inventory of Factors Affecting Successful Implementation and Sustainment) to Advance Context-Specific and Generalizable Knowledge of Implementation Determinants: Case Study of a Digital Contingency Management Platform

**DOI:** 10.21203/rs.3.rs-4912858/v1

**Published:** 2024-10-21

**Authors:** Andrea Jakubowski, Briana Patrick, Kira DiClemente-Bosco, Sarah Salino, Kelli Scott, Sara Becker

**Affiliations:** Montefiore Medical Center; Stanford University School of Medicine; Northwestern University Feinberg School of Medicine; Northwestern University Feinberg School of Medicine; Northwestern University Feinberg School of Medicine; Northwestern University Feinberg School of Medicine

**Keywords:** Contingency management, opioid treatment programs, opioid use disorder, implementation determinants, rapid qualitative analysis

## Abstract

**Background:**

Contingency management (CM) is the most effective treatment for stimulant use disorder but is underutilized by opioid treatment programs (OTPs), despite the high prevalence of stimulant use in this setting. As part of a state-wide initiative, we piloted a novel assessment, the Inventory of Factors Affecting Successful Implementation and Sustainment (IFASIS), to examine determinants of implementation of a digital CM platform across a set of OTPs. We describe how the IFASIS was used to elucidate both generalizable and context-specific implementation determinants, and to guide the provision of implementation facilitation.

**Methods:**

Six OTPs received a multi-level implementation strategy (including facilitation) to promote programmatic uptake of a digital CM platform. Pre-implementation, OTPs completed the IFASIS, a 27-item questionnaire that assesses both the valence (positive/negative) and importance of determinants across 4 domains: outside the organization, within the organization, about the intervention, and about intervention recipients. OTP staff completed the IFASIS as a team, identifying consensus ratings during recorded discussions. Transcripts of IFASIS recordings were analyzed using rapid qualitative analysis. Quantitative IFASIS results were aggregated into medians and ranges within and across organizations. Implementation facilitation meeting notes were analyzed to examine how the IFASIS was used to guide facilitation.

**Results:**

Quantitative ratings and qualitative feedback revealed common barriers to implementation of the digital CM platform, including a lack of sustainable funding sources, absence of external and organizational policies, insufficient higher-level leadership support, and mixed attitudes among staff members toward CM. Common implementation facilitators included enthusiasm and commitment among organization leadership and the perception that the digital CM platform would reduce the workload and burden on OTP counselors. The IFASIS was used to guide facilitation in several ways, including stimulating discussion about barriers and facilitators, brainstorming strategies to address barriers rated as “very important”, and identifying facilitators that could be harnessed as part of implementation efforts.

**Conclusions:**

The IFASIS identified important determinants of CM implementation in OTPs and was instrumental in shaping facilitation. The IFASIS may be a valuable assessment for the implementation science community to identify and address generalizable and context-specific implementation determinants.

## Background

The sharp rise in stimulant use among patients with opioid use disorder (1) has led national organizations to call for widespread implementation of contingency management (CM) in opioid treatment programs (OTPs) that dispense medication for opioid use disorder (MOUD) (2,3). Contingency management (CM), a behavioral intervention in which patients earn frequent incentives for meeting treatment goals, is the most effective treatment for stimulant use disorder and an effective adjunct to MOUD (4,5). Receipt of CM is associated with over twice the rate of abstinence compared to treatment as usual in randomized trials (4) and has large adjunctive effects when combined with MOUD (5). Recent data encouragingly indicate that about half of all OTPs report providing some form of CM (6), yet surveys of OTPs suggest that CM is not delivered with fidelity consistent with evidence-based guidelines (7,8).

Determinants (i.e., barriers and facilitators) of CM implementation in OTPs and other community organizations are well documented and span multiple (e.g., system, organizational, and provider) levels. Some established examples of CM determinants when delivered in-person include funding to cover the costs of CM incentives, organizational resources (i.e. staffing, time) to deliver CM with fidelity (9,10), and provider attitudes toward patients earning incentives (11). Widespread recognition of these determinants has led to efforts to increase CM accessibility via development of digital CM platforms that allow patients to remotely provide specimens for drug testing and receive monetary incentives electronically (12). Digital CM platforms have demonstrated acceptability to a wide range of patients, including those in rural areas, using multiple substances, and/or socioeconomically disadvantaged (13,14). Multiple studies in the United States have found that digital CM platforms are associated with higher rates of abstinence and engagement in treatment for alcohol, tobacco, and other substance use disorders relative to treatment as usual (15). Digital CM platforms have also been theorized to present a cost-effective alternative to in-person CM by reducing staff effort and patient burden (12,16–18).

Despite the potential of digital CM platforms to address well-established determinants, digital interventions introduce unique determinants, including reliance on internet/smartphone access, patient/provider concerns about data privacy and security, and the need for ongoing financial investment and information technology support as technology evolves (19). Yet, to date, little to no research exists on determinants of CM implementation using a digital platform. The current study aims to address this gap by elucidating such determinants across a cohort of OTPs given free access to a digital CM platform through a state-funded implementation initiative.

A key tension when attempting to elucidate implementation determinants – both for digital CM platforms in particular and behavioral health interventions in general – is reconciling the uniqueness of determinants in specific contexts with the scientific goal of producing generalizable knowledge. Generating context-specific, locally relevant implementation strategies requires a highly localized understanding of determinants, while producing knowledge that can help other OTPs necessitates approaches that reveal commonalities across programs. To address this tension, we used a novel, team-based assessment called the Inventory of Factors Affecting Successful Implementation and Sustainment (IFASIS) (20) in two complementary ways that varied in their intent: (a) to guide OTP-specific implementation facilitation (context-specific) and (b) to elucidate common determinants across OTPs (generalizable). In addition to generating knowledge about digital CM platforms, this manuscript serves as a case example of how the IFASIS can be employed both as a determinant assessment tool and an implementation facilitation guide, in a manner that advances both context-specific and generalizable knowledge.

## Methods

### Parent Trial

This study is embedded within a National Institute on Drug Abuse (NIDA)-funded research project called Maximizing the Implementation of Motivational Incentives in Clinics 2 (MIMIC2) (21). MIMIC2 is one of three coordinated research projects within The Center for Dissemination and Implementation at Stanford (C-DIAS), a P50 Center of Excellence designed to advance equitable access to evidence-based addiction treatments. MIMIC2 builds on a previous hybrid type 3 cluster-randomized trial, Project MIMIC, which evaluated the comparative effectiveness of two multi-level, theory-driven implementation strategies for CM targeting patient engagement across 28 OTPs (22).

MIMIC2 partnered with two Departments of Health (Rhode Island and Chicago, Illinois) to offer an optimized multi-level implementation strategy to help OTPs implement CM targeting stimulant use. The current analysis focuses on work done in partnership with the Rhode Island Department of Behavioral Healthcare, Developmental Disabilities & Hospitals (BHDDH) to support the state’s rollout of an evidence-based digital CM platform using opioid settlement funds. The MIMIC2 team led both the multi-level implementation strategy and the evaluation of the state-wide rollout. The comprehensive evaluation included the Project MIMIC measures and the full C-DIAS measurement battery. The focal assessment was the IFASIS, which was one of the C-DIAS common measures to evaluate contextual determinants. All MIMIC2 study procedures received approval from the Northwestern University Feinberg School of Medicine Institutional Review Board (Protocol STU00219088).

### Participating OTPs and Providers

BHDDH conducted outreach to administrators or directors of OTPs throughout Rhode Island to assess interest in participating in the state-funded initiative to implement a digital CM platform targeting stimulant use. Of the 13 OTP directors, six expressed interest, all of which were approved for participation by BHDDH. Table 1 presents information about the participating OTPs, including each organization’s for-profit status, patient census, and medications provided.

Once consented, OTP directors were asked to nominate staff from their OTP to receive implementation support. To be eligible for nomination, staff were required to have an active clinical caseload and be willing to receive implementation support over a 6-month period. Across OTPs, 5–18 staff were nominated per program.

### The Digital CM Platform

The digital CM platform was secure, HIPAA-compliant, and delivered up to $599 in incentives to each patient over a 6-month period. The platform served as an “off-the-shelf” complement to clinical care that required minimal ongoing engagement from the referring OTP. To enroll patients in the platform, OTP counselors could do one of the following: (a) call an enrollment “hotline” in the presence of the patient; (b) fill out an enrollment survey in the presence of or on behalf of the patient; or (c) give the patient the “hotline” and/or enrollment survey to self-enroll. As a standard part of the digital CM platform, all enrolled patients were sent a reloadable payment card, given a kit of saliva tests (either mailed to their home or picked up at their OTP), and given instructions to access the program via smartphone app or web browser. Patient access to the program began with a “Welcome Phase” requiring completion of baseline forms before beginning remotely delivered CM sessions in which patients received weekly random accountability messages instructing them to perform a saliva test within the next 60 minutes. Patients were required to video record themselves taking a saliva test; once the platform certified results, they received a financial incentive to a reloadable payment card. Patients earned additional incentives for completing self-paced skills modules and logging their attendance at the OTP until they reached the maximum incentive level. Referring OTP counselors had access to a provider portal where they could view client toxicology results and incentives earned to reinforce patient engagement in the platform.

### Implementation Strategy

To support implementation, the MIMIC2 team delivered the multi-level implementation strategy used in Project MIMIC, called the Science to Service Laboratory (SSL). The SSL has three components: didactic training, performance feedback, and external facilitation. Didactic training included a live training session with the administrators of the digital CM platform along with a pre-recorded educational video on CM principles. Performance feedback was provided via weekly newsletters to each participating OTP reporting on patient enrollment in the digital CM platform. Finally, of direct relevance to this analysis, the MIMIC2 team offered six monthly external facilitation sessions to participating OTPs between September 2023 and April 2024. Facilitation calls commenced within two weeks of the didactic training, were 30- to 60 minutes in length, and were led by doctoral-level implementation scientists. All OTP staff participating in the implementation initiative were invited to attend. Facilitation sessions were designed to systematically identify OTP-specific determinants of implementation and collaboratively brainstorm strategies to address barriers while capitalizing upon facilitators.

### The IFASIS

The IFASIS is a 27-item assessment that includes 4 major domains: Factors Outside Your Organization; Factors Within Your Organization; Factors about the Intervention; and Factors about the Person Receiving the Intervention. The IFASIS is administered as a team-based assessment, with team members encouraged to discuss each question to generate consensus ratings. The assessment can be self-administered by the team on paper or electronically via Qualtrics, or administered by a trained facilitator, who records the consensus ratings. For each question, team members choose a statement that most closely describes how the factor affects their program. Item scores range from 1–5, with 1–2 indicating the factor is a barrier with negative valence, 3 denoting that the factor is neutral, and 4–5 indicating the factor is a facilitator with positive valence. Team members then rate the importance of each factor to their implementation efforts on a scale of from 1 (not important) to 3 (very important).

At each OTP, organization leaders were instructed to invite 3–5 team members involved in implementation of the digital CM platform to participate in the IFASIS assessment. Team members were all aware that the IFASIS was being completed as part of the evaluation of the state-funded rollout of the digital CM platform. Five of the six OTP teams completed the IFASIS with a facilitator: team members were generally at their OTP, while the facilitator joined via Zoom. Facilitators were three female BA-level research assistants trained in IFASIS administration, all of whom had 1–3 years of clinical research experience. OTP staff had met two of the facilitators at the didactic training sessions. Four of the sessions had a second research staff member join to take process notes: one of these sessions was also observed by the IFASIS developer for training purposes. The five facilitator-led sessions (ranging from 30–62 minutes) were audio-recorded and transcribed verbatim. The sixth OTP completed the IFASIS independently, so a recording and transcript were unavailable. This OTP’s results were included in the quantitative but not the qualitative analysis.

### Quantitative Analysis

For each OTP, we plotted the consensus IFASIS score using a visual dashboard to simultaneously summarize which factors were facilitators, which were barriers, and which were perceived as most important. Across OTPs, we calculated the IFASIS score median and range for each item.

### Qualitative Analysis

To elucidate implementation determinants across OTPs, we employed rapid qualitative analysis. We chose this approach given the highly structured nature of the IFASIS and the pragmatic goal of the current study (identifying generalizable implementation barriers across OTPs). Our qualitative methods and results are reported in line with the Consolidated Criteria for Reporting Qualitative Research (COREQ) [see Additional file 1] (23). We used a summary template and matrix analysis approach (24) that included two female raters (AJ, a physician with rapid qualitative methods expertise, and BP, an MS-level research specialist with qualitative expertise), neither of whom was involved in the IFASIS or implementation facilitation sessions. AJ provided a brief orientation to rapid qualitative analysis to BP at the project start and feedback on BP’s completed summary templates (e.g., amount of information to include, use of quotes, etc.).

The two raters first drafted a summary template based on the IFASIS items with fields to describe reasons participants gave for IFASIS ratings and importance scores, other observations, including interview dynamics, and an interview summary. The raters finalized the summary template after dual analysis of one transcript. In total, three transcripts were dual analyzed. During the dual analysis phase, the raters met weekly and compared each other’s templates, discussed disagreements, and generated a master consensus template. The raters then single-analyzed the two remaining transcripts and compiled all finalized templates into a single matrix to facilitate comparisons across OTPs. Finally, the raters divided the IFASIS domains in the matrix and prepared summaries of each domain, highlighting major barriers and facilitators. Each rater reviewed the other’s written summaries and provided feedback until consensus was obtained about the most salient implementation determinants.

When summarizing qualitative results, we prioritize those IFASIS domains and subdomains that had data that provided explanations for or context to the numeric IFASIS scores. We include select numeric IFASIS scores in the qualitative results to emphasize the magnitude of the rating (i.e., subdomains where the valence was very low or very high) and provide further context. For subdomains in which participants provided numeric scores but had little discussion about reasons for scoring, we report numeric IFASIS scores for completeness.

### Review of Facilitation Notes

To examine the context-specific ways in which the IFASIS guided implementation facilitation, the two qualitative raters analyzed meeting agendas and minutes from the six months of implementation facilitation calls. Five of the six OTPs completed at least five monthly facilitation calls. The sixth OTP opted out of participating in the rollout of the digital CM platform immediately after completing the baseline IFASIS, citing staff concerns about patient privacy. In total, 28 agendas were available for analysis.

The two raters documented how the IFASIS assessment was used during each facilitation session and highlighted instances when specific implementation strategies were generated to address determinants documented in the IFASIS. This documentation was shared with research team members who had led facilitation calls (SJB, KS, KDB). Team members involved in the calls subsequently reviewed their personal notes and records, met as a group, and documented their consensus on how the IFASIS was used to guide facilitation and inform the selection of implementation strategies. When presenting results, we document the group consensus and share illustrative examples of how the IFASIS guided facilitation at different OTPs.

## Results

### IFASIS Participants

Across the five OTPs that recorded their IFASIS assessments, an average of 5 (3–7) staff participated per program, with a total of 28 staff members participating. Each OTP had at least one organizational leader participate, most commonly the OTP director or lead clinical supervisor. Each OTP also had multiple frontline staff participate, most commonly OTP counselors. Participant characteristics are presented in Table 2. Participating staff were predominantly female (86%), white (75%), and not Hispanic/Latino (82%), with either a Bachelor (39%) or Master’s degree or higher (29%). The average tenure of staff at their OTP was 5.3 years (*SD* 4.5), while the average tenure of staff in the substance use field was 10.1 years (*SD* 8.1).

### Quantitative Data Across OTPs

**Figure 1:**
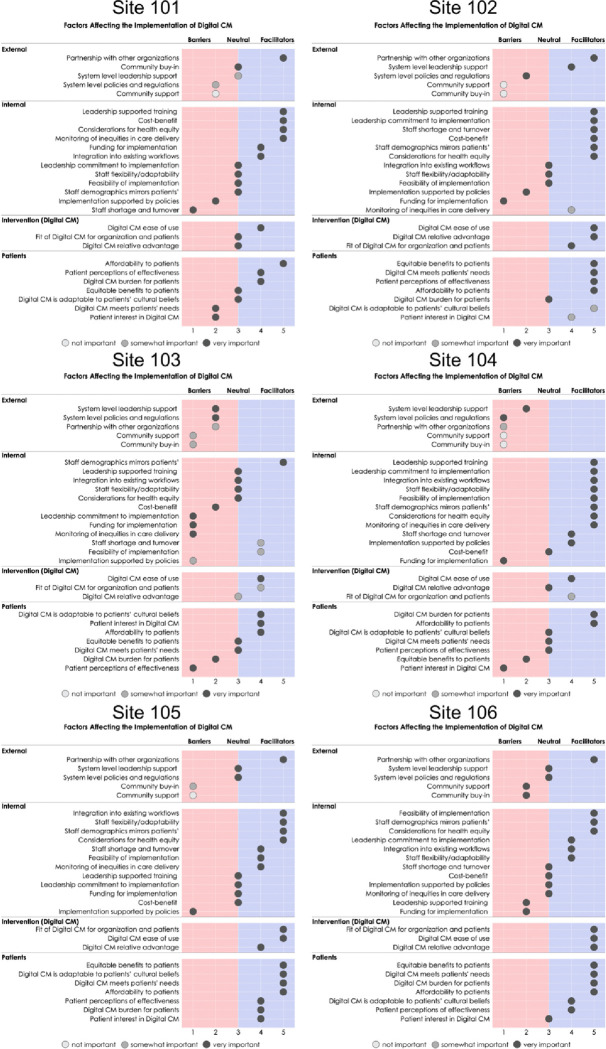
Visual graphic of results from the IFASIS conducted at baseline with each of the six OTPs. Each of the 27 items is listed, and the marker is placed at the consensus score. The further to the left the marker is placed, the more of a barrier it is perceived to be; the further to the right the marker is placed, the more of a facilitator it is perceived to be. The level of importance for each item is indicated by the shade of the marker, ranging from not important (white) to very important (dark gray).

Figure 1 presents the consensus IFASIS scores for each OTP. Each OTP received a visual dashboard that depicted determinants rated as facilitators (i.e., positive valence) in blue and those rated as barriers (i.e., negative valence) in red. The darkness of the circle indicated the importance rating assigned to the determinant. Items in the dashboard were listed in valence order; within each section, the most positive valence facilitators were listed at the top, and the most negative valence barriers were listed at the bottom.

Table 3 presents median IFASIS Ratings and Importance Scores across OTPs. The domain with the lowest ratings was *Factors Outside your Organization (External)*, with OTPs assigning particularly low ratings to external support, system-level policies, and support from and consultation with community organizations. The next lowest-rated domain was *Factors within your Organization (Internal)*, with particularly low ratings assigned to financial means to implement and organizational policies to implement.

The domain with the highest ratings was *Factors About the Intervention (Intervention)*, with participants consistently rating the fit, usability/complexity, and relative advantage of the digital CM platform as facilitators. The next highest-rated domain was *Factors About the Person Receiving the Intervention (Patients)*, with participants rating all items in the dimension (which span *Benefit to the Recipient and Recipient Needs and Values*) positively.

### Quantitative Data Across OTPs

We present qualitative data on implementation determinants of the digital CM platform organized by the five IFASIS domains. For simplicity, if a team completing the IFASIS endorsed a concept or gave a specific rating, we attribute the team’s input to the entire OTP (e.g., “five out of six OTPs reported fit was a barrier”).

#### Factors Outside Your Organization.

This IFASIS domain encompassed two sub-domains: external policies and community support.

##### External policies.

This sub-domain included two items about system-level leadership and system-level policies. Five of the six OTPs rated both system-level leadership and system-level policies as barriers or neutral. Interestingly, four of the OTPs acknowledged the existence of state-level support, and one identified the existence of a state-level champion to support the implementation of the digital CM platform. However, there was universal uncertainty about the extent of federal support and state and federal policies and regulations around CM (“If they exist, I don’t know what they are.” Site 102). Moreover, there seemed to be broad uncertainty as to whether the state-level leadership support would be sustained. All OTPs identified system-level leadership and system-level policies as very important.

##### Community support.

Three items comprised this sub-domain: community support; community buy-in; and partnerships with other organizations. The six OTPs universally rated community support and community buy-in as barriers or neutral (median ratings of 1.0), yet these determinants were generally rated as low importance because community organizations do not have “any say over our practices” or “don’t really support our treatment ideas anyway” (Sites 101 and 104). One OTP identified that the community had minimal information and education about CM. Only one OTP had begun introducing the digital CM platform to partner organizations but expressed concerns about receiving opposition, particularly from family treatment or drug courts. By contrast, four of the six OTPs viewed partnerships with other organizations as a facilitator of their implementation of the digital CM platform.

#### Factors within Your Organization.

This was the largest domain of the IFASIS, spanning five sub-domains: leadership, resources, organizational readiness, do-ability, and person-focused care.

##### Leadership.

This sub-domain included two items assessing support from OTP leadership to complete training and commitment from leadership to implement the digital CM platform. Two OTPs reported that perceived leadership support was strong and that leadership was supportive of training in CM. When reflecting upon leadership commitment, two OTPs described their leadership as having a “passion” or “pushing” for the digital CM platform but identified a lack of strategies or long-term plans to implement the platform in their setting (Sites 101 and 106). One OTP leader noted that they had not heard anything nor a “clear direction from corporate” about implementation of the digital CM platform (Site 103).

##### Resources.

This sub-domain had three items about staff retention, financial support, and cost-effectiveness. Staff retention was rated as a facilitator by four of the six OTPs (median rating of 4.0). These OTPs rated staff retention as high importance and acknowledged that it could affect implementation but noted that they were not currently experiencing shortages. One OTP noted that the digital CM platform made CM more feasible by reducing the clinical workload despite staffing challenges. Across OTPs, the funding for implementation item had the lowest ratings (median rating of 1.5). Four OTPs reported that without external funding, they would not be able to implement the digital CM platform, and one OTP expressed concern about sustaining it after the initiative ended. Just one OTP indicated that CM implementation was such a high priority that they would find a way to implement the platform, even without external support. Concerning cost-benefit, there was substantial variability in how OTPs perceived the digital CM platform: it was perceived as a facilitator by two OTPs, a neutral determinant by three OTPs, and a barrier by one OTP.

##### Organizational readiness.

This sub-domain contained three items about organizational policies, integration of CM into the workflow, and staff flexibility/adaptability. This sub-domain elicited substantial conversation across the participating OTPs. Organizational policies were viewed as a barrier by four OTPs (median rating of 2.0), and all six OTPs referenced the absence of organizational policies related to the digital CM platform. One OTP described policies as less important because they did not have policies for other innovations but were still able to implement them. Another OTP asserted that clear organizational policies from leadership would help with staff engagement in the implementation initiative.

Integration into the workflow received higher ratings (median rating of 4.0), but OTPs noted several challenges to integrating the digital CM platform into workflows. Staff burden was a theme that arose, with one OTP expressing concern about the need to manage “extensive” and “ever-changing” requirements (Site 102) and another noting that integration would be extremely difficult without a dedicated staff member responsible for the digital CM platform. Two other OTPs highlighted the importance of staff and leadership buy-in, with one OTP reporting that the most important predictor of whether they could incorporate the digital CM platform was “whether or not [staff] want to do this” (Site 104) and another OTP emphasizing the importance of having organization leadership monitor counselor use of the digital CM platform to promote integration of the platform into staff’s workflow (Site 103). Another OTP noted that the platform’s patients on stimulant use made integration challenging because the OTP did not treat patients with and without stimulant use differently. Finally, one OTP noted that having external support from the state was a major facilitator of their integration of the digital CM platform into the workflow.

The final item in this sub-domain was staff flexibility and adaptability. Three OTPs described staff as being very flexible or having adapted to the digital CM platform, while the other three OTPs rated this item as neutral (median rating of 3.5). Among those rating this factor as neutral, potential challenges mentioned included not all staff being adaptable or “open to extra work” (Site 102), small staff size making it more difficult to add on additional work, and lack of defined workflows being a barrier to staff adapting quickly to a new practice.

Do-ability and Person-centered care. The remaining two sub-domains, Do-Ability and Person-Centered Care, consisted of one and three items, respectively. The Do-Ability item assessed perceived feasibility of implementing the digital CM platform, whereas the Person-Centered care items assessed the extent to which staff demographics matched those of patients; the organization prioritized equity; and the organization measured patient demographics to assess inequities. All four of these items were rated highly (median ratings of 4.0 to 5.0) and generally viewed as facilitators within the existing OTPs.

#### Factors about the Intervention.

This IFASIS domain consisted of three sub-domains, each of which was only one item: fit, relative advantage, and ease of use. All three sub-domains had median ratings of 4.0, indicating that they were generally perceived as facilitators.

##### Fit.

Although five of the OTPs rated fit of the digital CM platform as a facilitator, two OTPs indicated that a minority of staff questioned the appropriateness of the digital CM platform or had not had enough experience to understand it. One OTP rated fit as neutral and expressed doubt in the platform, commenting “we are mostly not convinced” (Site 104).

Relative advantage: Three OTPs rated relative advantage as a facilitator, and three rated it as neutral. One of the OTPs that rated this item as a facilitator favorably compared the digital CM platform to take-home methadone bottles in terms of their ability to enrich usual care. Two of the OTPs that rated this item neutrally reported that the digital CM platform was complementary to, not better than, other services they offered.

Ease of use. All six OTPs rated the digital CM platform’s ease of use as a very high valence facilitator, and the OTPs generally rated this item as very important. This item had among the narrowest range of scores, with ratings ranging from 4.0 to 5.0.

#### Factors about the person receiving the intervention.

This domain spanned two sub-domains: Benefit to Recipient and Recipient Needs and Values.

##### Benefit to recipient.

This sub-domain contained only one item that assessed the extent to which the digital CM platform offered support to patients equitably. This item was generally viewed as a facilitator (median rating of 4.0). Despite the generally favorable ratings, OTPs identified several groups that they thought might not benefit from the platform, including (a) affluent patients who might not be incentivized by cash rewards (two OTPs); (b) non-stimulant users who are not eligible for the digital CM platform (two OTPs); (c) those without phones, without data on their phones, or who might be uncomfortable with technology (three OTPs); and (d) Spanish speakers who must use automated translation software that was viewed as sub-optimal (one OTP).

##### Recipient needs and values.

This sub-domain included six items that assessed the extent to which patients view the digital CM platform as adaptable to their cultural beliefs; able to meet their needs; burdensome; effective; something they would ask about; and affordable. Most comments were elicited by the items assessing whether the platform met patients’ needs and was something patients would ask about.

The platform’s ability to meet patients’ needs was generally rated favorably (median rating of 4.0): three OTPs rated this item as a facilitator, two rated it neutrally, and one rated it as a barrier. One of the OTPs that rated it as a facilitator indicated that “money is helpful for everyone” (site 102). Among the OTPs that rated this item neutrally, one indicated that the platform would not meet the needs of patients who were not eligible (e.g., those without stimulant disorder or a smartphone), and another expressed concern that the platform focused too much on “instant gratification” without emphasizing patient “accountability” (Site 104). The OTP that rated this item as a barrier noted that the digital CM platform did not (and could not possibly) meet all of patients’ needs.

Perceived patient interest was generally rated neutrally (median rating of 3.5). Several OTPs indicated that their ratings were driven by a lack of patient awareness, noting that patients were not asking about nor aware of the digital CM platform but would likely be interested when they learned about it. One OTP that had previously participated in Project MIMIC reported that patients supported CM. Another OTP noted that patients did ask about incentives in general and would likely appreciate the opportunity to use a digital CM platform.

The remaining items were generally rated as facilitators. OTPs rated the fact that the platform was free to patients as a very high valence, very important facilitator with a narrow range (median rating of 5.0, range from 4.0 to 5.0). OTPs also rated the platform’s adaptability and perceived effectiveness highly (median ratings of 4.0). OTPs did not perceive that patients would find the digital CM platform to be burdensome (median rating of 4.0), except potentially patients who had difficulty keeping appointments (two OTPs) or with transportation (one OTP).

### Use of IFASIS to Guide Facilitation

All six OTPs completed the IFASIS prior to initiation of the six monthly facilitation calls. In Month 1, the facilitator presented each OTP with their customized IFASIS dashboard (Fig. 1). Feedback was then solicited from OTP staff regarding whether the results were an accurate reflection of their early experiences implementing the digital CM platform. Using the visual dashboard, the facilitator first gained consensus around barriers and facilitators and then helped the OTP staff to prioritize which barriers should be addressed first, focusing on those with the most negative valence and rated most important. In Months 2 through 5, the facilitator engaged OTP staff in collaborative brainstorming to address those high-priority barriers while harnessing those facilitators with the most positive valence and rated most important. In Month 6, the facilitator conducted the IFASIS with each OTP again and shared feedback with each program about how the ratings and importance scores of determinants shifted over time.

Use of the IFASIS to guide facilitation sessions enabled a customized approach to the selection of implementation strategies. For instance, Site 103 identified leadership commitment to the implementation, funding for the implementation, and organization-level policies as important barriers to implementation of the digital CM platform. The facilitator guided the team in brainstorming several potential strategies to increase leadership commitment and support the development of organization-level policies, including several proposals for the state Department of Health funding the initiative: (a) providing higher levels of reimbursement for services offered by OTPs delivering CM; (b) providing incentives and recognition to OTP staff delivering CM; (c) requiring that leaders complete training in CM for an OTP to receive funding support; and (d) providing a state certificate or other form of recognition for that OTPs deliver CM. Another OTP identified staff retention as a strong, very important barrier (Site 101). This OTP proposed having a joint meeting and celebration between the state health department, OTP leadership, and the MIMIC2 study team to recognize those staff members who had been successful in implementing the digital CM platform as a means of increasing staff morale and promoting retention.

## Discussion

We used the IFASIS, a novel assessment that elicits both quantitative and qualitative data, to identify both generalizable and context-specific determinants associated with implementation of a digital CM platform. Findings from this study demonstrate how the IFASIS can be employed to assess determinants and guide implementation facilitation, and our case study in OTPs highlighted important barriers and facilitators unique to the digital CM platform.

We elicited several generalizable determinants across OTPs that aligned with well-established barriers to implementing face-to-face CM. Two of the most negative valence implementation barriers across OTPs included the absence of organizational policies and insufficient leadership support, consistent with research indicating that implementation climate (i.e., a climate in which the innovation is expected, supported, and rewarded) and leadership engagement are key determinants of CM implementation (25–27). During facilitation calls, OTP staff suggested that uptake of digital CM would increase if the digital CM platform were incorporated into existing workflows, incentivized by organizational leadership, and visibly led by leadership champions. These suggestions were well-aligned with established implementation strategies to promote the uptake of CM and other new practices(25,26,28)

Other generalizable barriers identified across OTPs that aligned with prior CM literature included lack of perceived community support and concerns about sustainability of funding. Regarding community support, OTPs have historically been heavily stigmatized (26,29), limiting the ability of staff to leverage local resources in service to their patients. OTPs could be supported in communicating with community members about CM and other services through community-focused implementation strategies such as education and stigma-reduction campaigns. Concerning funding, OTPs were nearly unanimous that sustaining the platform would be impossible without external support. Recent work has proposed that CM implementation could be funded via Medicaid, Substance Abuse and Mental Health Services Administration block grants, TRICARE (a funder of military and federal personnel), Indian Health Services, and opioid settlement funds (12,30). Reliable funding for CM – whether delivered face-to-face or via a digital platform - will be a crucial aspect of sustaining implementation efforts.

The IFASIS also identified a set of determinants across OTPs that were unique to the digital CM platform, including fit, relative advantage, ease of use, adaptability, and accessibility. In contrast to prior literature suggesting that providers have concerns about the effectiveness and fit of face-to-face CM models, the platform’s feasibility, ease of use, and effectiveness were all viewed as facilitators. These data confirm that a digital CM platform can potentially serve to address some of the barriers associated with face-to-face models. However, the digital CM platform was also associated with concerns about accessibility for non-English speakers, older adults, and individuals without phones or those with limited digital literacy. These results highlight the need for additional research on the cultural and linguistic appropriateness of digital CM (12,31) as well as the need to train OTP staff in strategies to access phones and data plans under the Affordable Care Act to allow patients without phones to access the digital CM platform (18,30).

Finally, the IFASIS identified a set of context-specific determinants that were used to guide implementation facilitation sessions. Variability in context-specific determinants across the six OTPs was notable. For instance, five of the IFASIS items had a range of scores from 1.0 to 5.0, highlighting the need to consider context-specific variability in multi-site implementation initiatives. Some of the items with the greatest variability in IFASIS scores included close partnerships with community organizations, commitment from internal leadership, and staff retention. These results suggest that different OTPs are likely to require different implementation strategies. An interesting future direction for the field could be to use tools such as the IFASIS to identify the most common set of determinants in a specific setting; develop modular, multi-component implementation strategies; and then tailor such strategies to the determinants identified in a specific context.

Results of this study must be interpreted in the context of limitations. First, OTPs occasionally misunderstood IFASIS questions and asked for clarification from the facilitator. Additional written instructions might help to limit facilitator influence. Second, as a new assessment, the IFASIS has not yet been evaluated for concurrent validity. This paper describes how the IFASIS can be used to elicit determinants but does not address the question of how the IFASIS would compare to existing guides such as the Consolidated Framework for Implementation Research (32). Third, the current study only examines determinants at the start of the implementation initiative. Future work will examine how these determinants changed with time.

## Conclusions

Despite these limitations, the current study provides a valuable illustration of how the novel IFASIS assessment can be employed to elicit implementation determinants and guide implementation facilitation. The IFASIS elicited a range of generalizable and context-specific barriers to implementing a digital CM platform, confirming that digital platforms face many of the same determinants as face-to-face models. Results also revealed determinants unique to the digital CM platform, which varied in their valence and importance across OTPs. The IFASIS assessment can support research teams and community partners in collaboratively assessing key determinants and selecting implementation strategies that will enhance the likelihood of sustained innovation implementation both across and within specific settings.

## Figures and Tables

**Table 1:** Characteristics of Each Participating Opioid Treatment Program (N = 6)

Characteristics	Organization IDs					
	101	102	103	104	105	106
Organization status (For vs. Non-Profit)	For-profit	For-profit	For-profit	For-profit	Non-profit	Non-profit
Patient census (Number)	550	605	625	502	850	350
Patients with stimulant use (%)	5–10%	20%	65–75%	50%	85%	40%
Patients dispensed methadone (%)	20%	85%	100%	90%	85%	91%
Patients dispensed buprenorphine (%)	75%	15%	0%	10%	15%	9%
Providers nominated for training (Number)	7	15	18	10	16	5
Nominated providers completing training (%)	100%	93%	94%	100%	100	100%

**Table 2:** Characteristics of Opioid Treatment Program (OTP) Staff Participating in Inventory of Factors Affecting Implementation and Sustainment (IFASIS) Assessments (N = 28)

OTP Staff Characteristics	N (% Staff)
Job type	
Direct clinical service provider	14 (50%)
Organization director or administrator	4 (14%)
Support staff	3 (11%)
Clinical supervisor	7 (25%)
Biological sex	
Female	24 (86%)
Male	4 (14%)
Race	
White	21 (75%)
Black of African American	3 (11%)
More than one race	3 (11%)
Unknown/Not reported	1 (3%)
Ethnicity	
Not Hispanic/Latino	23 (82%)
Hispanic/Latino	4 (14%)
Unknown/Not reported	1 (3%)
Education	
Some college, but no degree	3 (11%)
Associate's degree	5 (18%)
Bachelor's degree	11 (39%)
Master's degree or higher	8 (29%)
Unknown/Not reported	1 (3%)
Mean (SD)	
Average years of experience in the field	10.1 (8.1)
Average years with the organization	5.3 (4.5)

**Table 3:** Median Inventory of Factors Affecting Implementation and Sustainment (IFASIS) Valence and Importance Scores across Opioid Treatment Programs (N = 6) when Evaluating a Digital Contingency Management Platform

IFASIS Domains	Median Valence (Range)	Median Importance (Range)
**Factors Outside Your Organization (EXTERNAL)**		
**External Policies**		
Support from system-level leadership	3.0 (2–4)	3.0 (2–3)
System-level policies and regulations	2.0 (1–3)	3.0 (2–3)
**Community Support**		
Community support	1.0 (1–2)	1.0 (1–3)
Community buy-in	1.0 (1–3)	2.0 (1–3)
Partnership with other organizations	5.0 (1–5)	3.0 (2–3)
**Factors Within Your Organization (INTERNAL)**		
**Leadership**		
Leadership supported training	4.0 (2–5)	3.0 (3–3)
Leadership commitment to implementation	3.5 (1–5)	3.0 (3–3)
**Resources**		
Staff shortage and turnover	4.0 (1–5)	3.0 (2–3)
Funding for implementation	1.5 (1–4)	3.0 (3–3)
Cost-benefit	3.0 (2–5)	3.0 (3–3)
**Organizational Readiness**		
Implementation supported by policies	2.0 (1–4)	3.0 (2–3)
Integration into existing workflows	4.0 (3–5)	3.0 (3–3)
Staff flexibility/adaptability	3.5 (3–5)	3.0 (3–3)
**Do-ability**		
Feasibility of implementation and/or expansion	4.0 (3–5)	3.0 (2–3)
**Person Focused Care**		
Leadership and staff demographics mirror community	5.0 (3–5)	3.0 (3–3)
Prioritization and documentation of health equity	5.0 (3–5)	3.0 (3–3)
Monitoring of inequities in care delivery	4.0 (1–5)	3.0 (2–3)
**Factors About the Intervention (INTERVENTION)**		
**Fit**		
Fit for the organization and patients	4.0 (3–5)	3.0 (2–3)
**Usability/Complexity**		
Ease of use	4.0 (4–5)	3.0 (3–3)
**Relative Advantage**		
Advantages relative to the current approach	4.0 (3–5)	3.0 (2–3)
**Factors About the Person Receiving the Intervention (PATIENTS)**		
**Benefit to Recipient**		
Equitable benefits to patients	4.0 (2–5)	3.0 (3–3)
**Recipient Needs and Values**		
Adaptable to patients' cultural beliefs	4.0 (3–5)	3.0 (2–3)
Meets patients' needs	4.0 (2–5)	3.0 (3–3)
Patient perceptions of effectiveness	4.0 (1–5)	3.0 (3–3)
Burden for patients	4.0 (2–5)	3.0 (3–3)
Patient interest in the intervention	3.5 (1–4)	3.0 (2–3)
Affordability to patients	5.0 (4–5)	3.0 (3–3)

## Data Availability

The datasets generated and analyzed during the current study are available from the corresponding author on reasonable request.

## Abbreviations

BHDDH: Rhode Island Department of Behavioral Healthcare, Developmental Disabilities & Hospitals
CM: contingency management
HIPAA: Health Insurance Portability and Accountability Act of 1996
IFASIS: Inventory of Factors Affecting Successful Implementation and Sustainment
MOUD: medications for opioid use disorder
OTP: opioid treatment program
SSL: Science to Service Laboratory

## References

[1] Saloner B, Whitley P, Dawson E, Passik S, Gordon AJ, Stein BD. Polydrug use among patients on methadone medication treatment: Evidence from urine drug testing to inform patient safety. Addiction. 2023 Aug 1;118(8):1549–56. 37158468 10.1111/add.16180 PMC10330099

[2] Addiction Technology Transfer Center Network Contingency Management Task Force. SAMHSA Guidance for Implementation of Contingency Management Training and Technical Assistance [Internet]. 2024 Feb [cited 2024 Aug 14]. Available from: https://attcnetwork.org/products_and_resources/samhsa-guidance-for-implementation-of-contingency-management-training-and-technical-assistance/

[3] U.S. Department of Health and Human Services. Contingency Management for the Treatment of Substance Use Disorders: Enhancing Access, Quality, and Program Integrity for an Evidence-Based Intervention [Internet]. 2023 [cited 2024 Aug 14]. Available from: https://aspe.hhs.gov/reports/contingency-management-treatment-suds 39250570

[4] Ronsley C, Nolan S, Knight R, Hayashi K, Klimas J, Walley A, Treatment of stimulant use disorder: A systematic review of reviews. Vol. 15, PLoS ONE. Public Library of Science; 2020. 10.1371/journal.pone.0234809 PMC7302911 32555667

[5] Bolívar HA, Klemperer EM, Coleman SRM, DeSarno M, Skelly JM, Higgins ST. Contingency Management for Patients Receiving Medication for Opioid Use Disorder: A Systematic Review and Meta-analysis. Vol. 78, JAMA Psychiatry. American Medical Association; 2021. p. 1092–102. 34347030 10.1001/jamapsychiatry.2021.1969 PMC8340014

[6] Park TW, Shuey B, Liebschutz J, Cantor J, Anderson TS. Treatment Approaches for Opioid Use Disorder Offered in US Substance Use Treatment Facilities. JAMA [Internet]. 2024 Jul 11; Available from: https://jamanetwork.com/journals/jama/fullarticle/2820976 10.1001/jama.2024.11913 PMC11240225 38990551

[7] Rash CJ, Petry NM, Kirby KC, Martino S, Roll J, Stitzer ML. Identifying provider beliefs related to contingency management adoption using the contingency management beliefs questionnaire. Drug Alcohol Depend. 2012 Mar 1;121(3):205–12. 21925807 10.1016/j.drugalcdep.2011.08.027 PMC3243803

[8] Rash CJ, Alessi SM, Zajac K. Examining Implementation of Contingency Management in Real-World Settings. Psychology of Addictive Behaviors. 2019; 10.1037/adb0000496 PMC6980876 31343197

[9] Becker SJ, DiClemente-Bosco K, Rash CJ, Garner BR. Effective, but underused: lessons learned implementing contingency management in real-world practice settings in the United States. Prev Med (Baltim). 2023 Nov 1;176. 10.1016/j.ypmed.2023.107594 PMC10753028 37385413

[10] Khazanov GK, McKay JR, Rawson R. Should contingency management protocols and dissemination practices be modified to accommodate rising stimulant use and harm reduction frameworks? Addiction. 2024 Sep 1; 10.1111/add.16497 38627885

[11] Aletraris L, Shelton JS, Roman PM. Counselor Attitudes Toward Contingency Management for Substance Use Disorder: Effectiveness, Acceptability, and Endorsement of Incentives for Treatment Attendance and Abstinence. J Subst Abuse Treat. 2015 Oct 1;57:41–8. 26001821 10.1016/j.jsat.2015.04.012 PMC4561006

[12] Dallery J, Ives L, Knerr A. Toward an era of impact of digital contingency management in the treatment of substance use disorders. Prev Med (Baltim). 2023 Nov 1;176. 10.1016/j.ypmed.2023.107518 37080501

[13] Coughlin LN, Salino S, Jennings C, Lacek M, Townsend W, Koffarnus MN, A systematic review of remotely delivered contingency management treatment for substance use. Journal of Substance Use and Addiction Treatment. 2023 Apr;147:208977. 36804352 10.1016/j.josat.2023.208977 PMC10936237

[14] DeFulio A, Furgeson J, Brown HD, Ryan S. A Smartphone-Smartcard Platform for Implementing Contingency Management in Buprenorphine Maintenance Patients With Concurrent Stimulant Use Disorder. Front Psychiatry. 2021 Dec 7;12. 10.3389/fpsyt.2021.778992 PMC8688352 34950072

[15] Coughlin LN, Salino S, Jennings C, Lacek M, Townsend W, Koffarnus MN, A systematic review of remotely delivered contingency management treatment for substance use. Journal of Substance Use and Addiction Treatment. 2023 Apr;147:208977. 36804352 10.1016/j.josat.2023.208977 PMC10936237

[16] DeFulio A. Dissemination of Contingency Management for the Treatment of Opioid Use Disorder. Perspect Behav Sci. 2023 Mar 1;46(1):35–49. 37006603 10.1007/s40614-022-00328-z PMC10050478

[17] Higgins ST. Behavior change, health, and health disparities 2023: Contingency management for treating substance use disorders and promoting health in vulnerable populations. Prev Med (Baltim) [Internet]. 2023 Nov 1;176:107746. Available from: https://linkinghub.elsevier.com/retrieve/pii/S0091743523003262 10.1016/j.ypmed.2023.107746 PMC13222625 37898183

[18] Sweeney MM, Holtyn AF, Stitzer ML, Gastfriend DR. Practical Technology for Expanding and Improving Substance Use Disorder Treatment: Telehealth, Remote Monitoring, and Digital Health Interventions. Vol. 45, Psychiatric Clinics of North America. W.B. Saunders; 2022. p. 515–28. 10.1016/j.psc.2022.05.006 PMC9352538 36055736

[19] Graham AK, Lattie EG, Powell BJ, Lyon AR, Smith JD, Schueller SM, Implementation strategies for digital mental health interventions in health care settings. American Psychologist. 2020;75(8):1080–92. 33252946 10.1037/amp0000686 PMC7709140

[20] Chokron Garneau H, Cheng H, Kim JP, Abdel Magid M, Chin-Purcell L, McGovern MA. A Pragmatic Measure Of Context At The Organizational Level: The Inventory Of Factors Affecting Successful Implementation And Sustainment (IFASIS). Implementation Science Communications. 2024; 10.1186/s43058-025-00726-9 PMC12032751 40281591

[21] Becker SJ, DiClemente-Bosco K, Scott K, Janssen T, Salino SM, Hasan FN, Implementing contingency management for stimulant use in opioid treatment programs: protocol of a type III hybrid effectiveness-stepped-wedge trial. Implementation Science. 2023 Dec 1;18(1). 10.1186/s13012-023-01297-w PMC10498624 37705093

[22] Becker SJ, Murphy CM, Hartzler B, Rash CJ, Janssen T, Roosa M, Project MIMIC (Maximizing Implementation of Motivational Incentives in Clinics): A cluster-randomized type 3 hybrid effectiveness-implementation trial. Addiction Science and Clinical Practice. 2021 Dec 1;16(1). 10.1186/s13722-021-00268-0 PMC8505014 34635178

[23] Tong A, Sainsbury P, Craig J. Consolidated criteria for reporting qualitative research (COREQ): A 32-item checklist for interviews and focus groups. International Journal for Quality in Health Care. 2007 Dec;19(6):349–57. 17872937 10.1093/intqhc/mzm042

[24] Taylor B, Henshall C, Kenyon S, Litchfield I, Greenfield S. Can rapid approaches to qualitative analysis deliver timely, valid findings to clinical leaders? A mixed methods study comparing rapid and thematic analysis. BMJ Open. 2018 Oct 1;8(10). 10.1136/bmjopen-2017-019993 PMC6194404 30297341

[25] Becker SJ, Kelly LM, Kang AW, Escobar KI, Squires DD. Factors associated with contingency management adoption among opioid treatment providers receiving a comprehensive implementation strategy. Subst Abus. 2019 Jan 2;40(1):56–60. 29595403 10.1080/08897077.2018.1455164 PMC6163086

[26] Proctor SL. Rewarding recovery: the time is now for contingency management for opioid use disorder. Ann Med. 2022;54(1):1178–87. 35471190 10.1080/07853890.2022.2068805 PMC9045772

[27] Scott K, Jarman S, Moul S, Murphy CM, Yap K, Garner BR, Implementation support for contingency management: preferences of opioid treatment program leaders and staff. Implement Sci Commun. 2021 Dec 1;2(1). 10.1186/s43058-021-00149-2 PMC8088083 33931126

[28] Ramsey AT. Integration of Technology-Based Behavioral Health Interventions in Substance Abuse and Addiction Services. Int J Ment Health Addict. 2015 Aug 3;13(4):470–80. 26161047 10.1007/s11469-015-9551-4 PMC4492163

[29] Scott K, Murphy CM, Yap K, Moul S, Hurley L, Becker SJ. Health professional stigma as a barrier to contingency management implementation in opioid treatment programs. Transl Issues Psychol Sci. 2021 Jun;7(2):166–76. 34485617 10.1037/tps0000245 PMC8412039

[30] Becker SJ, DiClemente-Bosco K, Rash CJ, Garner BR. Effective, but underused: lessons learned implementing contingency management in real-world practice settings in the United States. Prev Med (Baltim). 2023 Nov 1;176. 10.1016/j.ypmed.2023.107594 PMC10753028 37385413

[31] Hammond AS, Sweeney MM, Chikosi TU, Stitzer ML. Digital delivery of a contingency management intervention for substance use disorder: A feasibility study with DynamiCare Health. J Subst Abuse Treat. 2021 Jul 1;126. 10.1016/j.jsat.2021.108425 PMC8197772 34116816

[32] Damschroder LJ, Hagedorn HJ. A Guiding Framework and Approach for Implementation Research in Substance Use Disorders Treatment. Psychology of Addictive Behaviors. 2011 Jun;25(2):194–205. 21443291 10.1037/a0022284

